# Case Report: Recurring Peritonitis and Dialysis Failure in a Toddler on Peritoneal Dialysis

**DOI:** 10.3389/fped.2021.632915

**Published:** 2021-03-04

**Authors:** Enas H. Mohammed, Sajimol Chandy, Abderrahman E. Kadhi, Ibrahim F. Shatat

**Affiliations:** ^1^Pediatric Nephrology and Hypertension, Sidra Medicine, Doha, Qatar; ^2^Pediatric Urology, Sidra Medicine, Doha, Qatar; ^3^Department of Pediatrics, Weill Cornell Medicine-Q, Doha, Qatar; ^4^Department of Pediatrics, Medical University of South Carolina, Charleston, SC, United States

**Keywords:** pediatric, peritoneal dialysis, end stage renal disease, peritonitis, foreign body

## Abstract

We report a case of a 2-year-old-boy with end stage renal disease (ESRD) secondary to posterior urethral valves (PUV) on peritoneal dialysis (PD). Our patient developed multiple episodes of peritonitis, refractory anemia and feeding intolerance over a 12-month-period. He was treated with multiple courses of intraperitoneal antibiotics. Despite being on high-calorie formula, he was slowly thriving. The feeding intolerance was attributed to past history of prematurity, gastro-esophageal reflux disease and ESRD co-morbidities. He had anemia resistant to erythrocyte stimulating agents and iron supplementation. His family received re-training and mastered the PD techniques. They reported no breach of the aseptic techniques. His workup which included multiple AP abdominal XR-plain films were read as unremarkable and showed the gastrostomy tube (GT) and the PD catheter in good position. He completed his antibiotic courses as prescribed after each peritonitis episode, peritoneal fluid cultures repeated after each treatment completion showed no growth. During the last peritonitis episode, our patient developed ultrafiltration failure. A cross-table abdominal XR was obtained to evaluate the peritoneal catheter position and showed an intra-abdominal foreign body. During surgery, a needle was laparoscopically removed from the ileum and the PD catheter was replaced. Subsequently, our patient's feeding intolerance and resistant anemia resolved. Finally PD was successfully resumed.

## Introduction

Every year, 1,500 children develop end stage renal disease (ESRD) in the USA (1), of whom one-third start on peritoneal dialysis (PD) and two-third start hemodialysis (HD) (2). PD is the predominant modality of renal replacement therapy (RRT) in infants and young children (2, 3) contributing to 96% of RRT in the 1st year of life (4).

PD provides multiple advantages compared to HD, to list some of which; PD is associated with better preservation of residual renal function and vascular access by avoidance of central venous access placement and its complications. PD when performed via cycler therapy at home and during the nighttime -as in most of the pediatric chronic PD therapies- minimizes interruption of daily activities, schooling and is arguably more physiological compared to HD, however requires substantial investment from the caregivers (5). On the other hand, children on PD are more likely to have hypoproteinemia and hypogammaglobulinemia as a result of peritoneal protein losses (3), increased dietary requirements, caregiver exhaustion and stress (5) and most importantly are more at risk for peritonitis. This is especially true in infants and younger children who are at higher risk of PD catheter distal cuff extrusion leading to possible leakage, exit site infection, and subsequent peritonitis (3, 6). Repeated and severe peritonitis may warrant PD catheter removal, cause dialysis membrane failure and in some cases, transition to hemodialysis (7–11).

In infants, pediatric and adult patients receiving PD, peritonitis is the most common dialysis-related complication (3, 12, 13). It is also a major contributing cause of mortality in infants on PD (3) and accounts for a variable proportion of mortality ranging from 5.9 to 33% in adults with an increased odds of death of up to four times after an episode of peritonitis (12).

In the adult population receiving PD, procedure-related infections accounted for the majority of peritonitis cases, with only 6% related to secondary intra-abdominal pathologies such as cholecystitis, appendicitis and bowel perforation (14). Peritonitis related to intra-abdominal pathology also had a higher mortality rate (15).

In patients receiving PD, peritonitis is defined by the International Society for Peritoneal Dialysis (ISPD) as the presence of two or more of (i) clinical features, i.e., abdominal pain and/or cloudy dialysis effluent; (ii) dialysis effluent white cell count above 100/μL or/with over 50% of polymorphonulcear leukocyte in the differential count; and (iii) identification of infective organisms from the dialysis effluent by Gram statin or culture (16).

## Case Presentation

Our patient is a 2 year-old boy, born preterm at 32 weeks of gestation with prenatal diagnosis of posterior urethral valve (PUV) and severely dysplastic kidneys. Shortly after birth, he underwent PUV resection with creation of bilateral ureterostomies and placement of a PD catheter and gastrostomy tube (GT).

Home Continuous cycling peritoneal dialysis (CCPD) was started at 2 months of age with good tolerance. Initially, our patient was thriving well and his anemia of chronic kidney disease was under control and responsive to treatment with erythrocyte stimulating agents and iron supplements. His PD prescription was 12 1-h cycles, Dianeal; Dextrose 1.36% (1.25%). He continued to have good urine output through his right ureterostomy (>1 mL/kg/h). PD ultrafiltration ranged between 200 and 300 mL/day.

At 12-months of age, our patient presented with fever, irritability and cloudy peritoneal fluid (PF). Physical examination revealed generalized abdominal tenderness. Catheter exit site was normal with no erythema or discharge. PF cell count showed >2,000 × 106 cells/L, 87% neutrophils. Culture was negative. He received a 14-day-course of intra-peritoneal (IP) cefipime treatment for culture negative peritonitis. Family underwent re-training and aseptic techniques during handling of PD catheter was re-enforced.

During subsequent follow up appointments, his parents reported occasional vomiting and irritability with every time they attempted to increase the feeding volume. This was attributed to gastroesophageal reflux disease and comorbidities related to prematurity and increased abdominal pressure because of concomitant night-time GT feeds and PD treatment. He was slowly gaining weight on high-calorie formula and overnight continuous feeding regimen. After consulting with the gastroenterology team and starting proton pump inhibitors, the family reported mild improvement in the feeding tolerance.

At 18 months of age, routine complete blood counts showed progressively worsening normocytic anemia (Hemoglobin 8.8–10.4 g/L) with low transferrin saturation (TSAT) below 20% despite adequate elemental iron supplementation up to 6 mg/kg/day, Darbepoetin 1 mcg/kg/week and good adherence to medication administration. Reticulocyte count was on the higher side (3%) with normal LDH, haptoglobin, peripheral blood smear, G6PD level, hemoglobin electrophoresis and coagulation profile. There were no complaints suggestive of gastrointestinal bleeding, however occasional dark stool was mentioned by the mother and was attributed to oral iron supplementation. The abdomen was soft on routine physical examination. Stool sample for detection of occult blood was not sent. Stool work-up for ova and parasites was negative.

**Figure 1:**
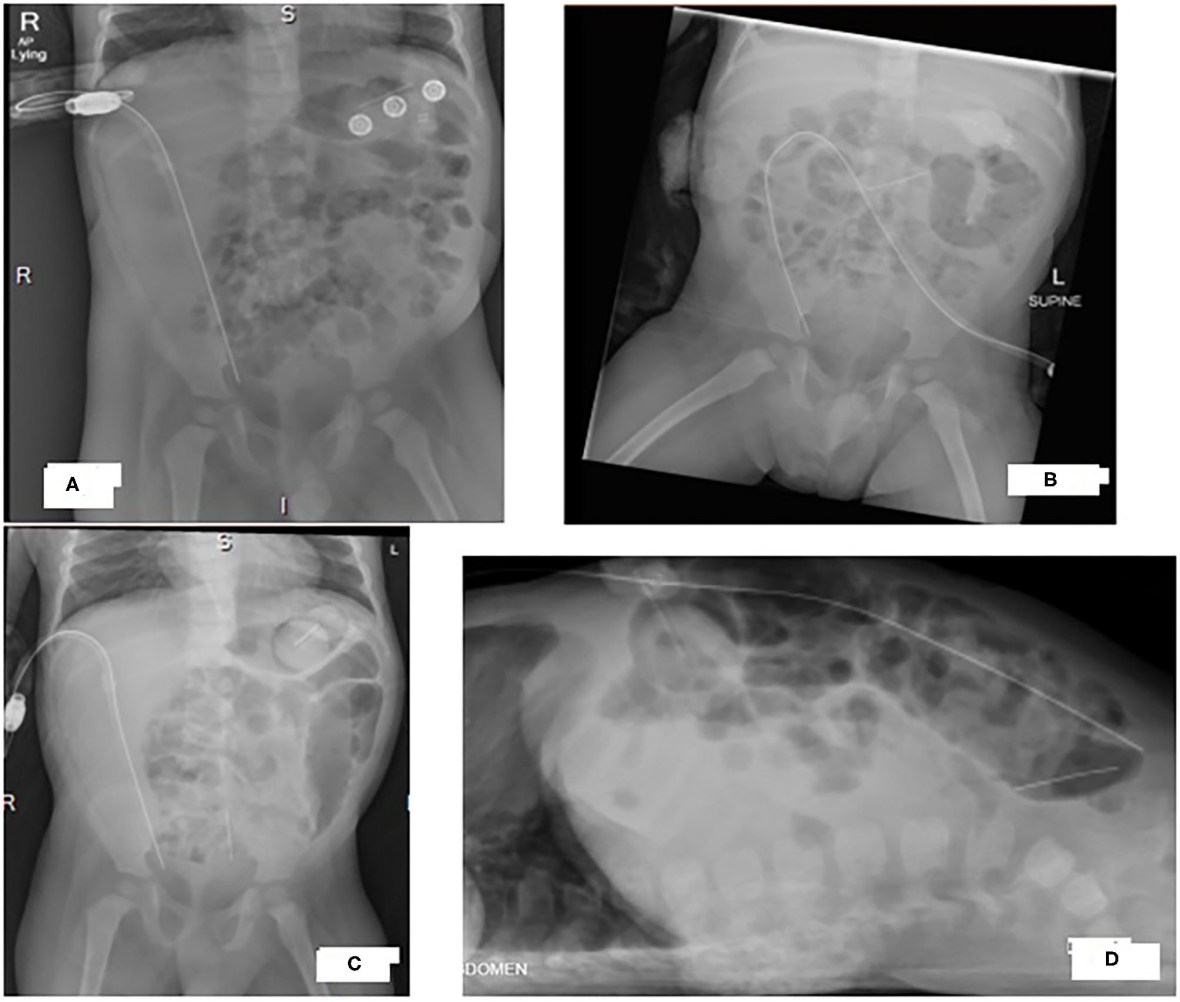
X-rays of our patient's abdomen showing progression of a radio-opaque horizontal thin object from the upper abdomen to the pelvis. **(A)** Antero-posterior view at 20-month, **(B)** Antero-posterior view at 22-month **(C)** Antero-posterior view and **(D)** cross table view at 24-month.

At 20 months of age, our patient presented with vomiting, diarrhea and fever. He was mildly dehydrated on examination, with erythematous tympanic membrane in the left ear. His abdomen was soft and PD fluid was clear. Work up for infectious etiology showed multiple organisms including rhino virus in respiratory secretions, adenovirus in stool and proteus in urine culture. PF cell count was normal and culture was negative. He was treated with oral Amoxicillin/clavulanic acid and intravenous (IV) hydration. During that admission, he was observed to have pain during the PD drain time. Anteroposterior (AP) abdominal X-ray (AXR) confirmed proper PD catheter position (Figure 1A). He was started on tidal PD. His PD prescription was 10 cycles in 11 h, tidal volume 90% with drain each 3 cycles, Dianeal; Dextrose 1.36% (1.25%), after which the family reported resolution of the pain.

At 23 months of age, our patient presented with fever, vomiting and decreased oral intake. He was febrile with generalized abdominal tenderness. Catheter exit site was clear with no redness or discharge.PF cell count >21,500 × 10^6^ cells/L with 93% neutrophils. He received a 14-day-course of IP Gentamycin treatment for ESBL Klebsiella pneumonie peritonitis.

Seven days after completing the IP antibiotic course, he was febrile again with abdominal pain and cloudy PD fluid. There was a delay in seeking medical advice as the family was traveling and by the time he presented to the emergency room, his PD catheter was not functioning (no outflow). He was started empirically on IV Meropenem. Administration of IP heparin with dialysis fluid led to mild fresh bleeding and pain was noted when mild negative pressure was applied via s a small syringe. There was no outflow from the PD catheter after that. AP and cross table AXR confirmed proper PD catheter position (Figures 1C,D). However, a foreign body (FB) was noted as a vertical thin metallic density overlying the sacrum with the appearance of a needle. Comparing the images to an AP AXR done 2 months before that (Figure 1B), the same dense object was seen more horizontally oriented in the upper abdomen. Cross table image was not done at that time and the mentioned object was overlooked as an artifact.

**Figure 2:**
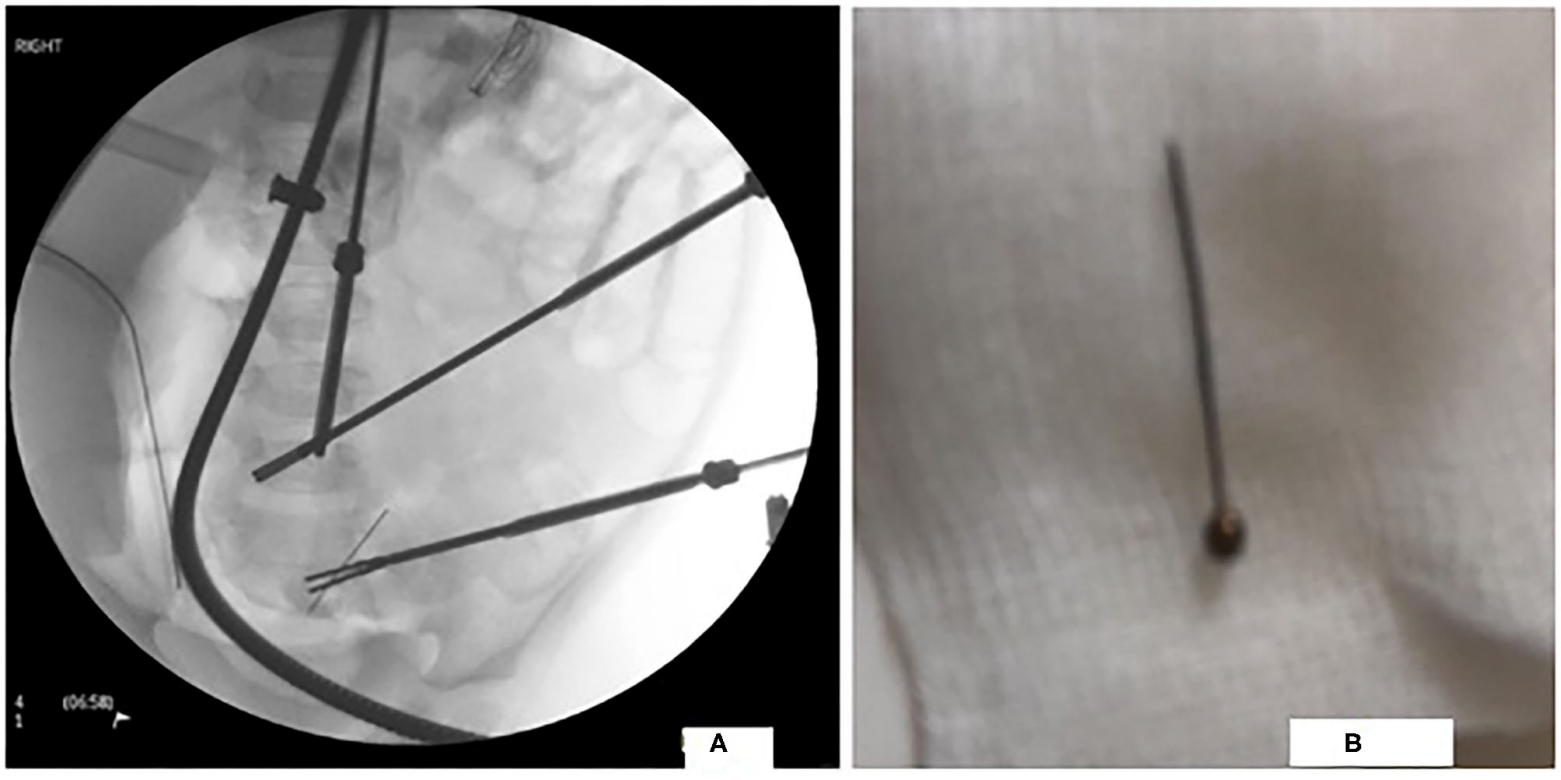
Laparoscopic image of the FB in the distal ileum in figure **(A)** and a photograph of the extracted FB in figure **(B)**.

Our patient underwent laparoscopic surgery. Multiple adhesions encapsulating the PD catheter were found between the ileum and the abdominal wall. A needle was extracted from the ileum through a mini-incision (Figures 2A,B). Adhesions were resected and PD catheter was replaced.

PD was restarted 2 months later. Currently our patient is thriving with good weight gain and his feeding intolerance resolved. Hemoglobin concentration is within normal range on treatment with erythrocyte stimulating agents (ESA) and iron supplements. By the time of writing of this report, 1 year has passed with no more episodes of peritonitis.

## Discussion

Our patient was started on PD therapy during his 1st year of life, which puts him at higher risk of dialysis-related complications especially peritonitis (3); Infants younger than 1 year have an annualized rate of peritonitis of 0.79 compared to children older than 12 years with peritonitis rates of 0.57 (3). Predisposing factors for peritonitis in children receiving PD include: age <1 year, the use of diapers, the presence of gastrostomy tube (17), all of which are present in our patient. However, our patient did well up to the 1st year of life without any episode of peritonitis. After that, he had culture negative peritonitis at the age of 1 year, feeding intolerance manifested as irritability with introduction of feeds and vomiting, anemia resistant to ESAs at 18 months of age, PD-related pain at 20 months of age, ESBL Klebsiella pneumonie peritonitis at 23 months and a relapsing peritonitis with PD failure at 24 months of age (Table 1).

**Table 1:** PD related complications in our patient.

**Age**	**PF cell count**	**Culture**	**AXR**	**Treatment**
12 months of age	>2,000 × 106 cells/L, 87% neutrophils	Negative	None	14-day-course of IP cefipime
20 months of age	Normal	Negative	AP only (Figure 1A) done to check for PD site(pain with PD drain time)	Oral antibiotics for UTI. No peritonitis.Tidal PD started.
23 months of age	>21,500 × 10^6^ cells/L- 93% neutrophils	ESBL Klebsiella pneumonie		14-day-course of IP Gentamycin
24 months of age	PD failure- No PD fluid taken		AP and CT (Figures 1C,D)	21-day-course of IV Meropenem (PD failure)

*PF, peritoneal fluid; AXR, abdominal X-ray; AP, antroposterior; IP, intraperitoneal; PD, peritoneal dialysis; UTI, urinary tract infection; ESBL, extended spectrum beta lactamase; CT, cross table*.

Repeated episodes of peritonitis and increased rates of PD complications should raise a red flag and should alert the treating clinician to peruse a more comprehensive work-up for secondary non-PD-related peritonitis (16) especially that our patient was peritonitis-free for the 1st year of his life.

According to peritonitis definition in (Table 2), our patient had two episodes of peritonitis, first was at 12 months of age, and the second was at 24 months of age followed by a relapsing peritonitis (16). Physical examination showed neither systemic manifestations of sepsis nor point tenderness, both of which are suggestive of a secondary cause of peritonitis (18). A root cause analysis including risk factors of PD-related peritonitis was done after each episode and peritonitis was attributed to poor adherence to infection control measures.

**Table 2:** Terminology related to peritonitis.

Recurrent peritonitis	A new episode of peritonitis occurring within 4 weeks of treatment following a prior episode with a different organism (16)
Relapsing peritonitis	A new episode of peritonitis occurring within 4 weeks of treatment following a prior episode with a same organism or with a negative culture (16)
Repeat peritonitis	A new episode of peritonitis occurring after more than 4 weeks of treatment following a prior episode with the same organism (16)
Secondary peritonitis	Peritonitis related to secondary intra-abdominal pathologies (14)

Frequent episodes of peritonitis may result in peritoneal sclerosis and thus peritoneal membrane failure. Patients with PD failure are frequently shifted to HD as a RRT modality. This change in modality will not only exhaust their vascular access but will also affect the child and parents' daily routines and school attendance (19). Moreover, peritoneal sclerosis was reported to cause intestinal obstruction, severe malnutrition and higher mortality in children and adults (19). Thus, carrying an extensive root-cause analysis and including *secondary non-PD-related* causes of peritonitis in the differential diagnosis is extremely important to both identifying the etiology and establishing a treatment plan (10).

Our patient also had feeding intolerance and resistant anemia, all of which have resolved after surgical extraction of the needle and release of the adhesions. He did not have a history of hematemesis, fresh bleeding from the rectum or melena. Occasional dark stools were described by the parents and were attributed to oral iron therapy. Laboratory investigation did not show any evidence of ongoing hemolysis.

Chronic blood loss and decreased iron absorption should be ruled out in patients with therapy- resistant iron deficiency anemia (20). Occult bleeding can be due to a number of gastrointestinal etiologies including parasite infections, peptic ulcers, polyps, hemangiomas and inflammatory bowel diseases (21). The improvement of our patient's anemia after the surgical extraction of the foreign body suggested the presence of chronic inflammation and possible intestinal occult blood loss as contributing factors.

FB ingestion most commonly occur between the ages of 6 months and 3 years and may present with vague non-specific symptoms requiring physicians to maintain a high index of suspicion. Most ingested foreign bodies pass through the GI tract without surgical intervention. However, serious complications, such as bowel perforation, obstruction and peritonitis-such as in our patient's case-can occur (22).

Three AXRs were done on different occasions (Figures 1A–C). All of which showed the FB in different sites of the abdominal cavity. It was only identified in the last setting when both AP and cross table images were done (Figures 1C,D). This argues in favor of routinely obtaining X-rays in two different planes to accurately diagnose and determine the exact shape and location of a FB (23, 24).

In conclusion, infants and toddlers undergoing PD who suffer from recurrent peritonitis, secondary causes of non-PD-related intra-abdominal pathology such as foreign body ingestion should remain in the differential diagnosis. The added diagnostic value of cross-table lateral abdominal x-ray was clear in our patient; arguably should be routinely added to AP AXR in peritoneal dialysis patients.

## Data Availability Statement

The original contributions generated for this study are included in the article/supplementary material, further inquiries can be directed to the corresponding author/s.

## Ethics Statement

Informed consent was obtained from the patients' family prior to presenting the case.

## Author Contributions

All authors contributed equally to this manuscript.

## Conflict of Interest

The authors declare that the research was conducted in the absence of any commercial or financial relationships that could be construed as a potential conflict of interest.
